# Academics’ experiences of work-life balance and work-life conflict in a familialist state: The case of Bosnia and Herzegovina

**DOI:** 10.12688/openreseurope.21781.1

**Published:** 2025-12-10

**Authors:** Sara Clavero, Caitriona Delaney

**Affiliations:** 1 Technological University Dublin, Dublin, Leinster, Ireland

**Keywords:** Work-life balance; Work-life conflict; Women academics; Bosnia and Herzegovina (BiH); Gender equality; Familialism; Thematic analysis.

## Abstract

**Background:**

This paper explores how women academics in Bosnia and Herzegovina (BiH) navigate work-life conflict and the main strategies they deploy to balance their work and personal lives. It examines the range of institutional support available to them and assesses their effectiveness in facilitating work-life balance and reducing work-life conflict.

**Methods:**

Drawing on 12 semi-structured interviews with academics at various career stages from public and private universities across BiH, this study captures personal narratives of managing work and caregiving responsibilities.

**Results:**

Thematic analysis revealed the respondents’ personal strategies to find work-life balance while trying to achieve their career goals, alongside the institutional factors acting as either barriers or enablers. Findings highlight the normalization of work-life conflict, often perceived as a ‘natural’ and ‘expected’ feature of academic life. The broader societal context and informal support networks -particularly collegial and familial–emerged as pivotal in shaping the respondents’ experiences and efforts to achieve work-life balance and to reduce work-life conflict.

**Conclusions:**

This paper adds to research on BiH and work life balance via its illustration of the multilayered experiences of academic life in BiH. Analysis of the interviews highlights the strategies employed by the respondents and the barriers they encounter, including gaps in institutional support to scaffold academics to achieve work-life balance.

## Introduction

This study was developed within the framework of the EDIRE project, funded by the European Commission under the Horizon Europe R&I Program (Grant Agreement Number 101060145). The project aims to strengthen research capacities and Equality Diversity and Inclusion (EDI) at the Sarajevo School of Social Science and Technology (SSST) and, more broadly, across Bosnia and Herzegovina (BiH). While work-life balance is widely acknowledged as a key factor in fostering gender equality, a survey conducted at SSST as part of the project revealed that the majority of respondents did not view work-life balance challenges as a major obstacle to their career advancement ( Bozzon *et al.*, 2017; Brough *et al.*, 2020; Lendák-Kabók, 2022; Rosa, 2022). This finding stands in sharp contrast to a substantial body of research highlighting the challenges academics face in achieving work-life balance and the adverse effects of work-related demands. Motivated by this discrepancy, the authors conducted an in-depth qualitative study, albeit with a small sample, across universities in Bosnia and Herzegovina. The aim was to gain a deeper insight into the lived experiences of academics in BiH and to contribute to the existing knowledge base by addressing this gap in the literature.

This paper draws from data collected from 12 semi-structured interviews conducted in October 2023 with academics at various career stages from universities in Banja Luka, Mostar, Sarajevo, and Tuzla. Data collection was guided by the following overarching research questions: How do academics in BiH experience work-life conflict and what strategies do they deploy to balance their work and personal lives? What barriers do they encounter in achieving work-life balance? What are the main gaps in the range of institutional support available? 

There is no universally accepted definition of work-life balance or work-life conflict. Work-life balance has been conceptualized in various ways: as the mere absence of conflict between work and personal life; as the degree of engagement and satisfaction an individual derives from both spheres; or as the successful negotiation and fulfilment of role-related expectations shared between the individual and their partners in work and family spheres ( Greenhaus *et al.*, 2003; Grzywacz & Carlson, 2007; O’Driscoll *et al.*, 2006). On the other hand, work-life conflict has been broadly defined as a tension between one’s professional responsibilities and personal and family life. It includes conflict on how time is spent as well as role conflicts, that is, feeling pulled in competing directions by the multiple roles and duties an individual must fulfil ( Jackson & Fransman, 2018; Jacob *et al.*, 2008; Li & Peguero, 2015; Netemeyer *et al.*, 1996). Considering this variety of definitions, in this paper, work-life balance and work-life conflict are understood as social constructs that are deeply ingrained in the organization of academic life. As social constructs, we understand these concepts as subjective and fluid (changing between individuals, contexts, and over time), while also agreeing with Rosa and other authors that how these concepts are both understood and experienced depends on one’s gender ( Rosa, 2022). In exploring how individuals attempt to balance an academic career with life outside of work in BiH, the study draws from and adds to the literature on both work-life balance and work-life conflict in the Higher Education sector in Europe.

The paper begins by providing brief contextual information on BiH, including a review of family policy instruments and a discussion of familialism. It then proceeds with a review of relevant literature, followed by a detailed account of the research methodology. The subsequent sections present and analyze the findings derived from the interviews, culminating in a summary of key insights and concluding reflections.

## Gender, paid labour and care in BiH

BiH may be considered an outlier regarding social policy, not only when compared with other former Yugoslav countries but also in relation to EU member states. In this section, we first examine gendered perceptions of paid labor and care, before turning to family policy instruments in the following section, which are discussed in the context of *familialism*. Our intention is to situate to work and other facets of life in BiH within a broader context that may shape experiences of work-life balance and work-life conflict in this country.

### Background

In BiH, formerly part of Yugoslavia, the current political structure consists of the decentralized Federation of Bosnia and Herzegovina (FBiH), the centralized Republic of Srpska (RS), and the District of Brcko (DB). After the war ended in the 1990s, benefit levels declined across all former Yugoslav countries, but the decrease was particularly pronounced in BiH and Croatia, both of which were severely impacted by the war ( Puljiz, 2008). The transition from socialism to capitalism, combined with processes of nation and state building, was accompanied by a weak gender equality agenda and limited fiscal capacities. welfare, labor, and economic reforms introduced during the post-war transition period often reinforced gendered socio-economic injustice ( Dobrotić, 2022; Kadić Abaz & Hadžic, 2020; Mulalić & Jeleškovic, 2022). In BiH, several legal instruments have been adopted to address women’s subordinate roles, such as the Law on Gender Equality (adopted in 2003). However, the implementation of these laws is often regarded as insufficient ( Kadić Abaz & Hadžic, 2020).

BiH society is widely characterized as both patriarchal and traditional ( Majstorović, 2011). After World War II, women became increasingly equal and emancipated. However, this trajectory shifted after the war in the 1990s, when patriarchal regimes and norms were reasserted. Reform proposals during this period often privileged traditional, gendered forms of labor within both the private and public spheres, signalling a return to the hetero-normativity and patriarchal structures of the pre-socialist era ( Dobrotić, 2022; Lai, 2020; Majstorović, 2011; Pavasović Trošt & Slootmaeckers, 2020). Within BiH families, women are typically regarded as the primary caregivers, while men are not expected to play a part in it ( Arslanagić-Kalajdžić *et al.*, 2023). As in many countries throughout the world, women engaged in paid employment frequently shoulder the bulk of care and household task, although women in BiH spend more time on care work than their counterparts in the EU overall ( Kadić Abaz & Hadžic, 2020; Obradović, 2021). Notably, perceptions of women’s domestic role are not confined to men but are also widely shared among women. Between 2015 and 2018, support for egalitarian gender roles declined, reflected in a 24% drop in citizens who believed that men and women should be equal ( Obradović, 2021). This trend suggests a broad consensus across genders regarding the traditionally prescribed each is expected to fulfil ( Kadić Abaz & Hadžic, 2020).

## Familialism

Familialism is a social system in which the family is considered as the primary locus of care and support ( Javornik, 2014; Leitner, 2003). It operates both at the policy level and within everyday practices, sustained by a tacit consensus at macro and micro levels that family members are the most appropriate caregivers ( Zaviršek & Fischbach, 2023).

Scholars distinguish between three forms of familialism: 1) ‘Familialism by default,’ where the state assumes that care is provided by the family; 2) ‘Prescribed familialism,’ in which legislation explicitly encourages family-based care, placing responsibility on relatives to provide and/or finance support; and 3) ‘Supported familialism,’ which recognizes the family as the main provider of care but introduces policies to assist caregivers, with the aim of promoting women’s labor market participation and advancing gender equality ( Saraceno, 2016). By contrast, defamilization refers to systems in which the state assumes primary responsibility for care provision ( Savinskaya, 2024). Defamilisation strategies include ‘supported defamilialisation’ through the market, where the state provides cash benefits to enable to purchase care services, and secondly, ‘defamilisation through public provision,’ where care services are directly provided by the state ( Verbakel *et al.*, 2023). Importantly, different forms of familialism and defamilization can coexist within a single welfare system ( Verbakel *et al.*, 2023). BiH, alongside other Western Balkan countries, is often regarded as a society in which traditional values prevail, with the family serving as the dominant provider of care for children, older people, and persons with disabilities ( Košarac & Kurteš, 2021; Obradović & Jusic, 2021). This reliance on family-based care has “serious consequences for gender equality in the country, as informal or family carers are usually women” ( Dobrotić & Stropnik, 2020).

## Family policy instruments

In BiH, family policy instruments can be grouped into three main categories: 1) child and family benefits, 2) parental leave policies, and 3) early childhood education and care (ECEC) and long-term care (LTC) ( Dobrotić, 2019). Child and family benefits are administered at the level of autonomous regions and remain among the least-funded schemes overall, accounting for only 2.5% of total social protection expenditure ( Dobrotić & Obradović, 2020).

With respect to parental leave, BiH experienced the sharpest post-1990 erosion of maternity rights among post-socialist Western Balkan countries ( Dobrotić, 2019). Decentralization in the late 1990s exacerbated territorial inequalities, with maternity benefits withdrawn in some areas ( Dobrotić & Obradović, 2020). Fathers (excluding those in the Brčko District) may access maternity leave after the 42nd or 60th day following childbirth, but only with the mother’s consent to transfer the leave, and only if both parents are employed and the mother returns to work while the father is on leave. This mother-centered approach, combined with entrenched traditional gender roles, reinforces gendered divisions of care and employment. For instance, in 2020, 256 women took maternity leave compared to just 11 men ( Agency of Statistics Bosnia and Herzegovina, 2022). Efforts to increase fathers’ participation began in 2000, largely in connection with EU negotiations and alignment with the EU parental leave directive ( Dobrotić & Stropnik, 2020).

ECEC provision has also declined since the socialist period, partly due to the destruction of facilities during the 1990s war. In 2019, there were approximately 7,000 fewer ECEC places than in the 1990s, leaving provision inadequate for the preschool population ( Dobrotić, 2019). Public ECEC services remain underdeveloped and inaccessible to many children across post-Yugoslav countries, with Slovenia as an exception, having introduced a legal entitlement in 1996. In BiH, by contrast, mandatory ECEC programs are not uniformly regulated across cantons ( Dobrotić, 2019). As a result, BiH ranks second to last globally in terms of the proportion of children enrolled in childcare services ( Arslanagić-Kalajdžić *et al.*, 2023).

Long-term care (LTC) policies place legal responsibility on families to provide for dependent members, including persons with disabilities and older relatives, while public services remain limited ( Obradović, 2021). Government-financed LTC allowances are insufficient, underfunded, and discriminatory. The scarcity of quality services means that families bear the primary burden of care, with institutional placement often becoming a last resort once home-based care is no longer manageable ( Obradović & Jusić, 2021). Unsurprisingly, this reliance on family-based provision disproportionately affects women, constraining their participation in paid employment and shaping their labor market experiences.

In sum, coherence across family policy instruments (child and family benefits, parental leave, ECEC, and LTC) is essential for balancing care, employment, and gender equality. Yet such coherence is currently absent in BiH, partly due to fragmented provision across state, regional, and employer levels ( Dobrotić, 2022; Moss & Deven, 2020). The lack of an integrated family policy contributes to underdeveloped care services and entrenched regional and occupational inequalities in maternity and other family benefits. As we argue later in this paper, this fragmented context directly shapes how academics in BiH experience and navigate work-life balance and work-life conflict.

## Work-life balance and work-life conflict in academic settings

Debates on work-life balance in academia have shifted from a focus on individual choice and investment to structural and organizational determinants ( Bozzon *et al.*, 2017). Neoliberal agendas and hegemonic work– centric models in universities significantly impact the work-life balance of academics. Within the neoliberal university, the everyday life of academics is determined by organizational practices and cultural norms modelled on the myth of the total availability of a solitary researcher ( Benschop & Brouns, 2003; Busso & Rivetti, 2014; Peroni *et al.*, 2015). This ideal is reinforced by precarious employment, competitiveness, high mobility, and expectations of hyper-productivity, all of which affect how academics plan and negotiate their private and professional lives ( Rosa, 2022). While both men and women encounter challenges with work-life balance in the context of the neoliberal university, women are more likely than men to experience work-life conflict ( O’Laughlin & Bischoff, 2005). Female faculty members often report time- and strain-based conflicts, noting that parenthood and caregiving responsibilities reduce opportunities for research, travel, networking, and daily academic work ( Bozzon *et al.*, 2017). Women are also more likely than men to experience frustration and guilt when forced to make difficult decisions, as care provision and family obligations are often perceived as detracting from full professional commitment ( Bomert & Leinfellner, 2017; Drew & Marshall, 2020). These dynamics reflect gendered organizational structures and entrenched societal gender stereotypes, with many women considering career interruptions or even leaving academia because caregiving responsibilities are still predominantly framed as women’s work ( Mulalić & Jeleškovic, 2022).

The struggle for women academics to achieve and sustain work-life balance is particularly acute in societies where women are expected to shoulder both paid labor and the entirety of domestic and care duties. BiH exemplifies such a context ( Hartig *et al.*, 2007; Lendák-Kabók, 2022; Pettigrew, 2021). Strategies used by female academics in BiH to achieve work-life balance and reduce work-life conflict include negotiating more equal sharing of caregiving within partnerships and adopting “double” or “split” shifts (alternating between paid employment and unpaid domestic labor) with heavy reliance on husbands and extended family ( Mulalić & Jeleškovic, 2022). These micro-level coping mechanisms, however, leave intact the organizational structures that underpin academic work. Many women academics in BiH perceive that their male colleagues advance more easily, given their comparatively limited domestic responsibilities, and report not being taken seriously due to prevailing societal beliefs that a woman’s place is in the home ( Mulalić & Jeleškovic, 2022). Institutional arrangements and family and social policies are therefore critical to making work-life balance attainable for both men and women, yet significant improvements remain necessary in BiH ( Mulalić & Jeleškovic, 2022).

Building on this body of research, the present paper examines the lived experiences of women academics in BiH as they attempt to reconcile academic careers with life outside of work. It also explores their perceptions of the institutional mechanisms required to reduce work-life conflict, facilitate career progression, and advance gender equality. To this end, we identified key discursive frames through analysis of narratives gathered in a series of semi-structured interviews, to which we now turn. 

## Methodology

The data for this study were collected through semi-structured interviews with 12 female academics from five universities in BiH, located across four cities: Banja Luka (one university), Sarajevo (two universities), Mostar (one university), and Tuzla (one university). Participants represented a range of disciplines and career stages (see Table 1 for respondent details).

**Table 1.** Respondent Information.

Respondent	Care Obligations	Marital Status	Gender	Career Stage
R1	Two young children and a sick mother	Married	F	Senior Teaching Assistant
R2	Two young children	Married	F	Senior Teaching Assistant
R3	Adult child	Married	F	Professor
R4	Two young children one with additional needs	Married	F	Professor
R5	Two older children and elderly parents	Married	F	Senior Teaching Assistant
R6	Yes	Married	F	Professor
R7	None	Single	F	Teaching Assistant
R8	Two young children	Married	F	Professor
R9	None	Single	F	Professor
R10	Two older children and a sick elderly mother	Married	F	Professor
R11	Two young two children	Married	F	Associate Professor
R12	Two young children	Married	F	Associate Professor

Semi-structured interviews were selected as the most appropriate method, as they enable the research to remain focused while allowing unexpected insights to emerge ( Adeoye-Olatunde & Olenik, 2021). This format also facilitates the exploration of participants’ experiences, understandings, and perspectives on specific topics ( McGrath *et al.*, 2019). An interview guide was developed following a review of the literature and the formulation of the study’s research questions.

Sampling was conducted using purposive snowballing, beginning with the authors’ professional networks in Sarajevo, who were asked to identify potential respondents. All interviews were conducted face-to-face, lasted between 40 minutes and one hour, and were recorded with participants’ consent. Transcripts were anonymized, with each respondent assigned a code (e.g., R1, R2), which is used throughout the Findings section.

 Written informed consent was obtained from all participants prior to data collection. Data were anonymized, and respondents are referenced in the findings using their assigned codes (e.g., R1). Ethical approval was granted by the institution’s Ethics Committee following submission of the research plans.

Data analysis followed Braun and Clarke’s six-phase approach to thematic analysis ( Braun & Clarke, 2006). This iterative process involves moving back and forth between phases, identifying patterns and themes, and allowing for analytic emergence. The analysis began with familiarization with the data, followed by the generation of initial codes, theme review and refinement, and concluded with the writing-up phase ( Braun & Clarke, 2006; Maguire & Delahunt, 2017). The lead author initiated the process, with the co-author independently reviewing the transcripts while remaining attentive to the research questions.

Having outlined the methodological approach, we now turn to the key findings.

## Findings

Guided by the research questions outlined earlier, our analysis identified four overarching themes: 1) societal context, 2) support, 3) expectations, and 4) strategies. While the normalization of overwork, the individualization of coping mechanisms, and reliance on informal support networks emerged as important issues for respondents, these patterns have already been extensively documented in the literature on academic labor, gender, and care. Accordingly, they are acknowledged here only briefly. Of greater significance, and central to this study’s contribution, is the interplay between institutional inflexibility and informal familial support, as well as the ways in which academic labor, gender, and familialism intersect in BiH.

The themes and sub-themes derived from the analysis are discussed in detail below. The discussion proceeds in two stages: first, we examine findings that align with previous studies, particularly those related to expectations and strategies; second, we turn to the more nuanced insights generated by this research, focusing on the societal context and forms of support that highlight the distinctive dynamics of BiH.

### Expectations and strategies

Although not entirely novel, respondents’ narratives revealed that work-life balance and work-life conflict are often perceived as inherent to academic life. Many participants noted that struggles in this regard should be “expected” when pursuing an academic career. For most, these challenges were so normalized that they tended to minimize their significance; some even paused during the interviews to reflect on the concepts themselves, with a few requiring clarification of the term work-life conflict.

Respondents frequently described “choosing” to work in ways that demand evenings and weekends, emphasizing that such commitments are part of academic life and widely understood as necessary for career progression. These accounts echo findings in existing research, which highlight the extent to which difficulties in achieving work-life balance are normalized within academia ( Fontinha *et al.*, 2019; Lantsoght, 2025).

In terms of strategies, several respondents reported feelings of guilt about not spending sufficient time with their children (R4) or noted that heavy workloads spilled into the domestic sphere, manifesting in frustration or anger directed at family members (R6). Nonetheless, many dismissed these challenges as manageable with proper organization. Strategies commonly mentioned included ensuring time for family meals and carving out space for leisure activities that provided relief from stress and a sense of personal time outside work.

R3 illustrates this approach:

My goal is not to work after 5 o’ clock in the evening. […] I try to finish everything, and what can be done tomorrow I will not do today. I joke with my husband when he starts to talk about work after 5 o’clock - I say ‘No! Now we go out’. We have a walk, a dinner, or something [and] we meet our friends and family. No work after 5 o’clock, if possible [...] that is my strategy (R3).

Similarly, R9 emphasized the importance of organization:

If you organize yourself properly, you’re not going to be in disbalance. […] The point is that I plan my work (R9).

Several respondents echoed this sentiment, suggesting that effective self-management makes work-life balance achievable. In sum, participants largely framed work-life balance as an individual responsibility, relying on personal strategies rather than questioning the organizational structures or policy frameworks that shape academic work. This individualization of coping mechanisms underscores the persistence of structural blind spots in addressing work-life conflict.

### Societal context

Societal context emerged as a thematically significant dimension of the study. Particularly salient is the social policy landscape in BiH, characterized by limited state provision of care, a strong reliance on family-based caregiving, entrenched patriarchal norms, and a complex political environment shaped by the legacy of the 1990s war and the decentralization of the state. The extracts below illustrate how these broader societal conditions shape respondents’ experiences of work-life balance and work-life conflict.

Several participants emphasized the importance of political connections in everyday life, ranging from access to childcare to securing employment opportunities. R4, for example, highlighted how political patronage influences academic careers:

When they have a position, it is a political decision who gets the job, [and] you need to have political connections which I don’t have. It’s very difficult (R4).

For R4, who works at a private university where flexible arrangements such as remote work or flexible hours are not available, the absence of political connections meant that moving to another institution with more supportive conditions was not a viable option. In this way, the broader political context directly constrained her ability to achieve work-life balance.

Similarly, R1 described how political ties affect access to childcare:

It’s awful, you need connections to get into daycare (R1).

This observation underscores how access to childcare—critical for reducing work-life conflict—is mediated by political networks rather than transparent policy implementation.

Respondents also reflected on the gap between family policies as written and their practical application. R4 described many policies as “non-implemented,” while R12 elaborated on the disconnect between rights on paper and their translation into practice:

[Policies] mostly exist in legal documents, we have this rights-based operation, but most rights are not translated into procedures. Even if they are, they 're not clear. They're very complex; you have a million documents, and they’re not readily available on a service-based level for a person to use (R12).

When discussing care responsibilities, respondents consistently noted that the prevailing expectation in BiH is for families to provide care for children and elderly relatives. They also observed that institutional services are often oversubscribed and of limited quality. R3’s reflection on elder care illustrates the cultural stigma attached to institutional provision:

In this society, we feel really bad about putting our elderly into the elderly home, because it's kind of frowned on here. Let’s say in other societies its normal, for us, it's not. I think it's a good thing because we believe that it's our duty to take care of our parents and our elderly, that’s nice. Many families really dedicate themselves to family care (R3).

Taken together, these accounts highlight how the societal context in BiH - marked by political patronage, weak policy implementation, and strong familialism - shapes the conditions under which academics attempt to reconcile professional and personal responsibilities.

### Support

Support emerged as a significant theme, with three salient sub-themes: institutional support, collegial support, and family support (including childcare and domestic labor). Respondents emphasized the importance of both formal and informal support, while also highlighting the consequences of its absence. A recurring pattern was the reduction of support—or lack thereof—to the individual level, framed as an inevitable aspect of academic life.

Many participants described feeling “lucky” to receive support, whether from colleagues, institutions, or family members. This framing suggests that support is perceived less as a right or entitlement and more as a discretionary favor. The repeated invocation of “luck” functions as a discursive tool that normalizes the absence of institutional structures and shifts responsibility for structural shortcomings onto individuals.

Institutional flexibility, such as the ability to work from home, was repeatedly mentioned as critical. Respondents working in universities that offered flexibility, particularly when combined with collegial support, reported greater capacity to balance professional and caregiving responsibilities. For example, R1 described how institutional flexibility, supportive colleagues, and a husband who shares domestic duties made work-life balance feel achievable, enabling her to meet both professional and caregiving obligations (children and a sick mother). Similarly, R8 noted that flexibility allowed her to “jump in” when unexpected situations arose, such as caring for a sick child.

By contrast, R4 worked in a university where flexibility was unofficial and contingent on personal disclosure. She described feeling uncomfortable when required to justify leaving early for caregiving responsibilities, recounting a dean’s dismissive comment:

Every time I have to leave work early, I have to ask my dean [for permission]. Once she said something like, ‘Don’t you have a husband?’ I didn’t say anything, but I wanted to say, ‘You know, he makes most of the money. Why are you asking me that? This is a private thing. Then, I have to explain why I have to leave. I don’t always feel like sharing that information. If I have to go, I have to go. It’s usually a last resort that I have to go. It’s difficult. So, I don’t do it anymore. (R4).

This extract illustrates how the absence of institutional flexibility not only exacerbates work-life conflict but also undermines employees’ sense of being valued. Onsite childcare was also identified as a potential institutional support, though respondents noted its absence. As R1 explained:

University day-care would help a lot, but that isn’t happening, but they do have a plan for that, which would help really a lot. It would be great to have (R1).

Collegial support was consistently described as vital. R1 emphasized the importance of supportive colleagues during periods of intense caregiving responsibilities:

We have really amazing colleagues who they’re very supportive. It has been really chaotic for the last two years, with my mother being sick and with a two-year-old. But it’s always okay to call somebody and ask them to fill in for my classes […] I think that’s very important. People have been amazing, which is great, because, with my mom, it’s not something that has just happened […] it started eight years ago, people were really supportive then as well. I am well aware that we have really nice colleagues, and it helps a lot. (R1).

Family support was also central. Respondents described parents caring for grandchildren, adult children supporting elderly parents, and spouses sharing domestic responsibilities. R8 noted that in BiH, family care is the default solution after maternity leave:

In Bosnia, the first solution, when you have a child, after maternity leave is if you have healthy parents, that they care for the children when you are at work. (R8).

R9 talks about returning to work after having a baby, thus illustrating how care and family support intersect. Reliance on family support for childcare points towards the prevalence of familialism in BiH and is evident in the quote below:

My parents help me a lot; actually, a support network was kind of developed especially with my second child as I had to return [to work] after giving birth after only 42 days, because we had quite a low salary (R9).

Spousal support was often framed in terms of “luck,” with women describing themselves as “managers” of household responsibilities even when husbands contributed. R10 explained:

I have to handle the household and take care of my children. Yes, we try to divide the chores equally. But, in my opinion, it’s never equal, at least I’m the manager (R10).

R11 echoed this sentiment, noting that while her husband was supportive, she remained responsible for organizing and managing domestic life:

I was lucky enough to have a good husband […] We definitely help each other. I’m not the only one doing all the housework. But I am organizing and managing (R11).

Similarly, R1 described her husband as “amazing” and an equal parent, while acknowledging that this was unusual in BiH’s patriarchal context:

I have an amazing husband. I really do […] he’s really like an equal parent, which to be honest is not really the most common thing in Bosnia. We are kind of a patriarchal society […] it’s mostly women’s job to take care of the kids, even though she’s working and so on. (R1).

Finally, R5 highlighted the importance of parental support in enabling her to balance professional and family responsibilities:

I need to balance my education, my stressful work, and my family because at the same time I want to be a good mother, to keep my family together […] When my children were smaller, they attended pre-care and post-care until I finished my work. I then arranged everything with my husband. I was also very lucky to have my parents, and they are in good health. They help me a lot, but without them, I couldn't make it at all (R5).

In sum, respondents’ reliance on “luck” to describe support from colleagues, institutions, and family members underscores the normalization of inadequate formal support structures. This discursive framing shifts responsibility for work-life balance and conflict onto individuals, obscuring the structural and institutional dimensions of the problem. This issue will be explored further in the Discussion section.

## Discussion and conclusions

The analysis of the interviews illustrates the multilayered experiences of academics in Bosnia and Herzegovina (BiH). Respondents’ accounts shed light on how they encounter work-life balance and work-life conflict, the strategies they employ, and the barriers they face—particularly the absence of institutional support structures that could scaffold academics in achieving balance.

A salient theme was the expectation that work-life conflict, or at the very least struggles with balance, is an inherent feature of academic life. Many respondents described working late into the evening and on weekends as congruent with the demands of academia. Everyday life for academics was thus shaped not only by professional expectations but also by broader societal norms.

Exploring strategies, barriers, and institutional gaps provides a window into the wider societal context in BiH, where academics’ coping mechanisms are constructed within a framework of familialism, patriarchy, and political complexity. Respondents’ strategies often involved working a “double” or “split” shift—combining paid labor with unpaid domestic responsibilities. While this phenomenon is not unique to BiH, it is particularly pronounced in societies where women are expected to shoulder both professional and domestic duties ( Kadić Abaz & Hadžić, 2020).

The data highlight how work-life balance and conflict are frequently viewed as “natural” features of academic careers. Respondents often reduced the challenge to individual responsibility, emphasizing personal strategies such as time management, organization, or sacrifice of leisure. Several noted that if one is unwilling to work long hours, sacrifice free time, and prioritize career over personal life, academia may not be a suitable path. This framing underscores how work-life balance is individualized rather than understood as a structural issue.

The repeated invocation of “luck” further illustrates this dynamic. Respondents described support from family, colleagues, or institutions as a matter of fortune rather than entitlement. This discursive framing conceals the demanding nature of academic work and shifts responsibility away from universities and the state, leaving individuals to manage balance largely on their own.

Strategies employed by respondents ranged from careful organization and workload management (R10, R8) to reliance on family networks for childcare and elder care. Yet these strategies were often insufficient in a context where societal expectations reinforce women’s double burden. For example, R9 described working evenings and weekends to “top up” tasks left unfinished during the day, while still ensuring time for family.

Barriers to balance were deeply embedded in BiH’s patriarchal and traditional social fabric. Respondents pointed to the dominance of family-based care, limited childcare provision, and a decentralized political system that complicates policy implementation. Institutional support was notably absent: respondents emphasized the lack of on-site childcare and the limited availability of flexible work arrangements. R4’s account of being denied flexibility at her university illustrates how institutional cultures can exacerbate conflict and undermine employees’ sense of value.

Informal support - whether from colleagues, spouses, or extended family - was described as crucial. Yet even when husbands were supportive, many women positioned themselves as “managers” of the household, responsible for organizing and overseeing domestic life. This reliance on informal networks reflects the dominance of familialism in BiH, where family care is normalized and institutional provision remains scarce.

A striking finding is the extent to which respondents have internalized expectations of academic life. Many described struggles with balance as a “natural” part of the profession, reinforcing the idea that academic work is never complete and rarely confined to standard working hours. This normalization of imbalance highlights the need to interrogate how structural and cultural factors shape academics’ lived experiences.

This study contributes to research on BiH by demonstrating how work-life balance and conflict are negotiated within a context of weak institutional support, entrenched gender norms, and familialist social structures. Future research could explore why and how work-life conflict has become normalized and expected within academia, and how these dynamics intersect with broader societal conditions. Comparative studies across the Western Balkans and European Union member states would further enrich the literature on work-life balance, work-life conflict, academia, and gender.

## Ethics and consent

This study was approved by the Research Ethics Committee of Technological University Dublin (approval number: REIC-21-181). Written informed consent was obtained from all research participants prior to data collection.

## Data Availability

The data underlying this article are available in Zenodo at https://doi.org/10.5281/zenodo.17643044 under the CC-BY 4.0 ( Clavero & Delaney, 2025). The dataset includes anonymised and de-identified transcripts of interviews conducted for gathering data for the study.
