# Supplementary material for: Academics’ experiences of work-life balance and work-life conflict in a familialist state: The case of Bosnia and Herzegovina
Source: Open Res Eur. 2026 Feb 18;5:380. Originally published 2025 Dec 10. [Version 2] doi: 10.12688/openreseurope.21781.2 (PMC12917355; doi:10.12688/openreseurope.21781.2)
Supplement: Peer review history

# Reviewer report: Reviewer response for version 2

**DOI:** 10.21956/openreseurope.24981.r69944

**Published:** 2026-02-19

**Authors:** Paweł Larionow

**Affiliations:** 1 University College of Professional Education, Wrocław, Poland

For Authors: The manuscript has been improved according to my recommendations. Thank you for these revisions.

If applicable, is the statistical analysis and its interpretation appropriate?

Not applicable

Is the study design appropriate and is the work technically sound?

Partly

Is the work clearly and accurately presented and does it engage with the current literature?

Partly

Are the conclusions drawn adequately supported by the results?

Partly

Are sufficient details of methods and analysis provided to allow replication by others?

Partly

Are all the source data and materials underlying the results available?

Partly

Reviewer Expertise:

psychometrics, psychological assessment, health psychology

I confirm that I have read this submission and believe that I have an appropriate level of expertise to confirm that it is of an acceptable scientific standard.

# Reviewer report: Reviewer response for version 1

**DOI:** 10.21956/openreseurope.23560.r66048

**Published:** 2026-01-13

**Authors:** Rachel Palmen

**Affiliations:** 1 Universitat Oberta de Catalunya, Barcelona, Spain

This paper provides an interesting overview of Academics' experiences of work-life balance and work-life confilct in Bosnia & Herzegovina. The paper could be improved with the following minor revisions. 

 1) Include the key data that is available on gender (in)equalities in Bosnia & Herzegovina from the latest She Figures 2025. This could be a quick overview of the main findings (specifics of Bosnia & Herzegovina) and a quick overview of B&H in a comparative context. 

 2) I don't understand the sentence "benefit levels declined across all former Yugoslav countries" - does this mean state funded benefits? This needs to be explained better. 

 3) In the discussion on familialism - it might be interesting to locate this discussion within the gender regime literature.  

 4) There could be a bit more detail about the coding process and how the main thematic areas were generated. 

 5) I don't think the title "Societal context" sufficiently encompasses the three main concepts that this area deals with. Maybe Socio-Political Context is better.

If applicable, is the statistical analysis and its interpretation appropriate?

Not applicable

Is the study design appropriate and is the work technically sound?

Yes

Is the work clearly and accurately presented and does it engage with the current literature?

Yes

Are the conclusions drawn adequately supported by the results?

Yes

Are sufficient details of methods and analysis provided to allow replication by others?

Partly

Are all the source data and materials underlying the results available?

Yes

Reviewer Expertise:

Gender equality in research and innovation from a comparative European perspective.

I confirm that I have read this submission and believe that I have an appropriate level of expertise to confirm that it is of an acceptable scientific standard.

# Reviewer report: Reviewer response for version 1

**DOI:** 10.21956/openreseurope.23560.r66046

**Published:** 2026-01-06

**Authors:** Paweł Larionow

**Affiliations:** 1 University College of Professional Education, Wrocław, Poland

**Please find the comments:**

**Abstract**

 Please indicate that females the sample comprises females.

**Introduction**

 Starting the introduction with mentioning a specific project is uncommon.

 The 2nd paragraph in the introduction should be moved below to the lat part of the introduction.

 Overall, please restructure the parts before Methodology.  For instance, the aim of the study was duplicated in different places of the introduction. Please see academic guidelines of how to structure a scientific paper.

**Methodology**

 Please specify when the study was conducted.

 Please specify the date and number of the ethical approval as well as the name of the committee.

 The age of respondents was not indicated.

 Please indicate how interviews were conducted (in what form, where, how recording was done).

**Results**

 In my view, results are concise and clear.

**Discussion and conclusions**

 In the discussion, there are only 1 reference. Please clearly contrast your findings with prior literature.

 Please present separate conclusions.

 Please create a table with main topics and provide issues, practical implications or potential solutions. Using a table, please synthesize the information about experiences of work-life balance and work-life conflict. This will definitely improve the readability of this paper.

 Limitations and future directions should be provided.

**Ethics**

 The data of approval should be provided.

 Consent to publish verbatim statements of the respondents should be provided.

If applicable, is the statistical analysis and its interpretation appropriate?

Not applicable

Is the study design appropriate and is the work technically sound?

Partly

Is the work clearly and accurately presented and does it engage with the current literature?

Partly

Are the conclusions drawn adequately supported by the results?

Partly

Are sufficient details of methods and analysis provided to allow replication by others?

Partly

Are all the source data and materials underlying the results available?

Partly

Reviewer Expertise:

psychometrics, psychological assessment, health psychology

I confirm that I have read this submission and believe that I have an appropriate level of expertise to confirm that it is of an acceptable scientific standard, however I have significant reservations, as outlined above.
